# Seroprevalence and trends of transfusion transmitted infections at Harar blood bank in Harari regional state, Eastern Ethiopia: eight years retrospective study

**DOI:** 10.1186/s12878-018-0115-2

**Published:** 2018-09-15

**Authors:** Zelalem Teklemariam, Habtamu Mitiku, Fitsum Weldegebreal

**Affiliations:** College of Health and Medical Sciences, Department of Medical Laboratory Sciences, Haramaya University, P.O. Box 235, Harar, Ethiopia

**Keywords:** Transfusion transmitted infections, Hepatitis B virus, Hepatitis C virus, Human immunodeficiency virus, Syphilis, Blood bank, Harar, Eastern Ethiopia

## Abstract

**Background:**

The use of unscreened blood exposes the patient to many transfusion transmitted infections including Hepatitis B Virus (HBV), Hepatitis C virus (HCV), Human Immunodeficiency Virus (HIV), and syphilis, among others. Thus, blood transfusion demands for meticulous pre-transfusion testing and screening. Trends of transfusion transmitted infections are important to take appropriate measures on blood bank services. Therefore the aim of this study was to assess seroprevalence and trends of transfusion transmitted infections at Harar blood bank in Harari regional state, Eastern Ethiopia from 2008 to 2015.

**Methods:**

A retrospective cross-sectional study was employed to review blood donors’ history and laboratory tests records from November 16–December 31, 2017. All records of blood donors having vividly documented history and laboratory tests were reviewed by data collectors. All data were entered into EPI data version 3.1. It was exported and analyzed with Statistical Package for the Social Sciences version 16 soft ware.

**Result:**

A total of 11, 382 blood donors’ history and laboratory tests records were reviewed. Majority of them were males (82.6%), 57.6 % were in the age group of 17 to 25 years and 99.9% donors donated blood for the first time. The overall seroprevalence of transfusion transmitted infections (HBV, HIV, HCV and syphilis combined) was found to be 6.6%. The prevalence of HBV, HIV, HCV and syphilis were found to be 4.4%, 0.6%, 0.8% and 1.1%, respectively. The trend in prevalence of syphilis and HCV was statistical significant by year (p< 0.05). Those donors in the age group of 26–35 years (AOR: 2.1; 95% CI: 1.2,3.6), 36–45 years (AOR: 4.1; 95% CI: 2.4,7.1) and greater than 46 years (AOR:4.6; 95% CI: 2.3,9.1) were more likely to be infected with syphilis compared to the age group of 17–25 years. Male were more likely to be infected with HBV (AOR: 1.9; 95% CI: 1.4, 2.5) than females.

**Conclusions:**

The magnitude of transfusion transmitted infections was lower than the previous studies conducted in Ethiopia. However, the decline in trends of transfusion transmitted infections has not been significant for some pathogens. Therefore, strict adherence with the criteria of preliminary blood donor selection should be implemented to reduce the amount of blood being withdrawn from transfusion after collection and screening.

## Background

Blood transfusion services (BTS) are transfusion of blood components. This saves millions of patients from death and morbidity each year worldwide [1]. There were reports that a quarter of a million mothers deaths globally due to obstetric bleeding. Furthermore, anemia causes 15% of child death in Africa [2]. Ethiopia is a country with high number of maternal mortality [3], motor accident and population non-immune to malaria. All the above cases required safe blood transfusion [2]. Blood transfusion corrects different conditions of patients like anemia, deficiency of plasma clotting factor, thrombocytopenia, hypoalbuminia and other [4].

It is known that blood can be vehicle for number of blood pathogens. Transfusion of unscreened blood to the patient has risk of acquiring Transfusion Transmitted Infections (TTIs) like Hepatitis B Virus (HBV), Hepatitis C Virus (HCV), Human Immunodeficiency Virus (HIV), syphilis, malaria and other. Despite uncertainty in the exact rates of transmission, blood transfusion is still considered to be a contributing mode of viral transmission in parts of Africa. Proportion of new HIV infections might ranges from 5 to 10% [5]. Similarly, 12.5% of patients at risk of post transfusion hepatitis [6]. HBV is the highly known potential infectious virus which is associated with complications like cirrhosis, portal hypertension and hepatocellular carcinoma [7].

Several Studies had been conducted to assess the prevalence and trends of TTIs among blood donors in different parts of the world. They found out an increase or decrease of trends TTIs [8–13]. Thus, blood transfusion units of each health institutions should have major role to screen, monitor and control TTIs. This practice also give some clues about the magnitude of TTIs in healthy populations’ [1, 8].

Selection of blood donors with low TTIs risk was followed by effective laboratory screening in major work of blood transfusion units [14, 15]. These activities have been extremely effective but transmission of diseases still occurs. Because of the inability of the laboratory test to detect those persons with an infection in the window period, lack of budget for all standard laboratory for TTIs testing and trained manpower, presence of immunologically variant viruses, presence non-seroconverting silent carriers, laboratory testing errors and poor quality control of laboratory tests [9, 16–18].

There were few studies on the seroprevalence and trends of TTIs among blood donors in Ethiopia, which found with variable findings [11, 19–23]. A study conducted among Ethiopian blood donors in 1995 showed that the seroprevalence of HIV-1, syphilis and HBV was 16.7%, 12.8% and 14.4%, respectively [23]. In another retrospective study conducted in Gondar from January 2003 and December 2007 showed a seroprevalence of HIV, HBV, HCV and syphilis of 3.8%, 4.7%, 0.7%, and 1.3% respectively. And significantly declining trends of seroprevalence of HIV, HCV and syphilis was observed [19]. There was no published report about the prevalence and trends of TTIs among blood donors in Harari region. Hepatitis B Virus (HBV), Hepatitis C Virus (HCV), Human Immunodeficiency Virus (HIV) and syphilis are the four major Transfusion Transmitted Infections (TTIs) which have been routinely screened from all blood donated at Harar Blood Bank. Therefore we sought to assess the trends in donor seroprevalence of HBV, HCV, HIV and syphilis over an 8 year period (2008–2015).

## Methods

### Study area and period

The study was conducted from November 16- December 31, 2017 in Harar blood bank, Harari Regional State. Harar town is the capital city of Harari People Regional state, which is one of the most historical towns, located in the eastern part of Ethiopia. It is found at 525 km east of Addis Ababa, the capital city of Ethiopia. The blood bank was established in 1976/77 by Ethiopian Red Cross society. It has been collecting blood from donors by undergoing campaign in different institutes, routinely screens the collected blood for the presence of four major TTIs (HIV, HCV, HBV and syphilis) and provides screened blood to recipients in need at hospitals in the regional state. In addition, they prepare different blood components including platelet, and plasma as well as providing voluntary counseling for all blood donors after testing their blood sample.

### Study design

A retrospective cross-sectional study was employed to review blood donors’ history and laboratory tests records.

### Sample size and sampling techniques

The sample size for prevalence and trends of transfusion transmitted infections was determined by using single population proportion formula considering the seroprevalence of HIV, HBV, HCV and syphilis found as 3.8%, 4.7%, 0.7%, and 1.3%, respectively from blood donors at Gondar University Teaching Hospital, Northwest Ethiopia [19]. Sample sizes for estimating seroprevalence of HIV, HBV, HCV and syphilis in the above-mentioned study were 351, 430, 67 and 123, respectively. Even if the largest sample size from the previous study (i.e. 430) would have been taken as the final sample size for this study. But, for greater point estimate and power of the analysis, all (*n* = 11,382) blood donors’ history and laboratory tests records were reviewed at Harar Blood bank from 2008 to 2015.

### Method of data collection

Blood donors were either volunteers, or replacement remunerated or mobile (donates blood during campaign). In the blood bank, all blood donors are required pass through a panel of questions on previous illnesses and medical conditions and physical examination for blood donation eligibility according to the national blood donation criteria. The eligibility criteria were age between 17 and 65 years, body weight > 45 kg, no history of high-risk sexual behavior and practice, blood transfusion, jaundice, hepatitis, surgery, and hypertension, and current fever. The medical and socio-demographic histories of the donors were recorded on each individual blood donor history record and venous blood was collected from each donor by laboratory technologists following standard procedures.

All blood donors’ history and laboratory tests records documented at Harar Blood bank from 2008 to 2015 were reviewed by trained nurses working in the blood bank. Information such as age, sex, marital status, occupation status, residence, blood donor types (replacement (family, remunerated), volunteer and mobile (donates blood during campaign)), frequency of donation and laboratory examination results were reviewed.

### Laboratory examination

All blood donors samples were tested for HBsAg, anti-HCV and HIV using Wantai AiD™ HBsAg Enzyme Linked Immuno Sorbant Assay (ELISA),Wantai AiD™ anti-HCV ELISA and WANTAI HIV 1 + 2 Ag/Ab ELISA test kit respectively developed by Beijing Wantai Biological Pharmacy Enterprise Co., Ltd. China Laboratory diagnosis. And for syphilis: using DIALAB ELISA developed by Nora Kampitsch, MSc, India. The anti-syphilis Ab ELISA test is a one-step enzyme immunoassay for the qualitative detection of antibodies to *Treponema Pallidum* in human serum or plasma. All the positive blood samples tested were repeated in duplicate before labeling them as seropositive by the same tests. Confirmatory test about active infection was not performed.

### Operational definition

**Voluntary donors**: donor gives blood of his or her own free will and receives no payment, either in the form of cash or in kind.

**Replacement donors**: donors of blood who replace blood used by their relatives or friends from blood bank stocks.

**Mobile donors:** a donors who gave blood only once during blood donation campaign made at people gathering in school, in community or other institution for their own activities.

**Transfusion Transmitted Infections (TTI**): infectious agents including HBV, HIV, HCV and Syphilis.

### Data quality control

Those blood donors’ history and laboratory tests records with complete study variables were included in this study. Training was given to two nurses before actual data collection in order to assure the quality of data. Each data collected by data collectors was checked for completeness at end of each day by principal investigator. Preset of data collection tool was made at Dire Dawa blood bank.

### Data analysis

Data were coded, entered into EPi data version 3.5.1 and exported to Statistical Package for the Social Sciences (SPSS) version 16 for analysis. To define the prevalence of TTIs, the number of TTI-positive donations during each year was divided by the total number of blood donations that year with 95% confidence interval (CI). The prevalence across different years and socio –demographic variables was compared using the Chi-square test. Regression analysis was done to assess the association between each TTIs with some socio–demographic variables. The Cochran–Armitage trend test (Z) was used to determine any significant trends in the rates of infected donations over time. Statistical significance was set at *p* < 0.05.

## Result

In this study, a total 11,382 blood donors’ history and laboratory tests records were reviewed. The mean age of the blood donors were 27 years with standard deviation of ±8.8 and range of 18–65 years. Majority of them were male (82.6%) in sex and in the age group of 17–25 years (57.6). Most of the blood was collected from mobile donors (56.0%), and those who donated blood for first time (99.9%). Majority of the blood donors had blood group O (45.1%) and Rhesus factor (RH) positive (93.4%) (Table 1).

**Table 1:** Characteristics of blood donor who donated blood from 2008 to 2015 in Harari regional state blood bank in Eastern Ethiopia

Variables	No (%)
Sex	
Male	9403 (82.6)
Female	1979 (17.4)
Age	
17–25	6555 (57.6)
26–35	2934 (25.8)
36–45	1402 (12.3)
≥ 46	490(4.3)
Occupation	
Farmer	1081 (9.5)
Military	1956(17.2)
Government employed	1896 (16.7)
Daily laborer	163(1.4)
Driver	222(2.0)
Factory worker	154 (1.4)
House wife	110(1.0)
Student	4079(35.8)
Private employed	1259(11.1)
Unemployed	132(1.2)
Other	330(2.9)
Donor type	
Mobile	6376 (56.0)
Replacement	4089(35.9)
Voluntary	917 (8.1)
Number of donation	
First time	11,369 (99.9)
Repeated	13 (0.1)
Blood group	
A	3151 (27.7)
B	2434 (21.4)
AB	661 (5.8)
O	5136 (45.1)
RH type	
Positive	10,634 (93.4)
Negative	748 (6.6)
Donor address Region	
Harari	6217(54.6)
Oromiya	3214(28.2)
Dire Dawa	919(8.1)
Somali	887(7.8)
Other(Addis Ababa, Afar, Amahar, Benishangaul, Southern Nations, Nationalities, and Peoples', Tigray)	144(1.3)

### Seroprevalence of transfusion transmitted infections

The overall seroprevalence of TTIs (total seroprevalence of HBV, HIV, HCV and Syphilis) was 6.6% (95% CI: 6.2–7.2%). The seroprevalence HBV, HIV, HCV and syphilis were 4.4%, 0.6%, 0.8% and 1.1%, respectively. A total of 0.2% (24/11382) blood donor had coinfections. From whom, HBV-syphilis (45.8%) (11/24) and HBV-HIV (20.8%) (5/24) coinfections were the dominant ones (Table 2).

**Table 2:** Prevalence of co-infections of HIV, HBV, HCV and syphilis among blood donors from 2008 to 2015 in Harari regional state blood bank in Eastern Ethiopia

Coinfections	No (%)
HBV/ HIV	5 (0.04)
HBV/HCV	4 (0.04)
HBV/Syphilis	11 (0.1)
HCV/syphilis	2 (0.02)
HIV/syphilis	2 (0.02)
Total (*n* = 11,382)	24 (0.2)

### Trend of HIV, HBV, HCV and syphilis

The prevalence of HBV, HIV, HCV and syphilis was the highest in the year 2008 (6.3%), 2008 (1.2%), 2012 (3.1%) and 2015(2.6%), respectively. The prevalence of HBV and HIV was not statistical significant different by year. However, the prevalence of HCV declined in most years, but it started to increase by the year 2009 and 2012. While, the prevalence of syphilis declined in most years except the highest record in 2015. The difference in prevalence of syphilis and HCV was statistical significant by year (*p* < 0.05). But, the overall difference in the prevalence in TTIs ((total seroprevalence HBV, HIV, HCV and Syphilis), was not statically significant by year (*p* > 0.05) (Table 3).

**Table 3:** Trends of seropositivity of HBV, HIV, HCV and Syphilis among blood donors from 2008 to 2015 in Harari regional state blood bank in Eastern Ethiopia

Year	Total screened	HBV positive No(%)	HIV positive No(%)	HCV positive No (%)	Syphilis positive No(%)
2008	253	16(6.3)	3 (1.2)	2 (0.8)	4 (1.6)
2009	239	10(4.2)	1(0.4)	3 (1.3)	–
2010	581	28(4.8)	5 (0.9)	4(0.7)	7(1.2)
2011	984	52(5.3)	8 (0.8)	5(0.5)	3 (0.3)
2012	1146	41(3.6)	3 (0.3)	35(3.1)	1(0.1)
2013	1549	76 (4.9)	8(0.5)	6(0.4)	1 (0.1)
2014	2523	110 (4.4)	10 (0.4)	23(0.9)	–
2015	4107	167 (4.1)	25 (0.6)	14(0.3)	107 (2.6)
Total	11,382	500(4.4)	63 (0.6)	92 (0.8)	123 (1.1)
*P* value of linear regression trend		0.101	0.361	0.001	0.000

### Seroprevalence and associated factors of transfusion transmitted infections

Blood donors who were in the age group of 26–35 years, 36–45 years and greater than 46 years were two times (AOR:2.1; 95% CI: 1.2,3.6), 4 times (AOR: 4.1; 95% CI: 2.4,7.1) and more than four times (AOR: 4.6; 95 CI: 2.3,9.1) more likely to be infected with syphilis than those who were in the age group of 17–25 years. Regarding occupational status, students (AOR: 0.2; 95% CI: 0.04, 0.8) and private employees (AOR: 0.2; 95% CI: 0.03, 0.9) were 80% less likely to be infected with syphilis than non-employees. Replacement blood donors were 70% less likely to be infected with syphilis than voluntary donors (AOR: 0.3; 95% CI: 1.6, 6.7) (Table 4).

**Table 4:** Characteristics of blood donors associated with HIV and Syphilis sero positivity from 2008 to 2015 in Harari regional state blood bank in Eastern Ethiopia

Characteristics	HIV positive No (%)	Crude odd ratio 95% CI	Adjusted odds ratio 95% CI	Syphilis positive No (%)	Crude odd ratio 95% CI	Adjusted odds ratio 95% CI
Sex						
Male	51/9403(0.5)	1		108/9403(1.1)	1	1
Female	12/1979(0.6)	0.9(0.5,1.7)	0.6(0.3,1.1)	15/1979(0.8)	1.5(0.9,2.6)	0.9(0.5,1.5)
Age						
17–25	21/6555(0.3)	0.5(0.1,1.4)	2.2(0.7,7.3)	34/6555(0.5)	1	1
26–35	28/2934(1.0)	1.3(0.4,3.7)	0.8(0.3,2.3)	36/2934(1.2)	2.4(1.5,3.8)	2.1(1.2,3.6)**
36–45	10/1402 (0.7)	0.9(0.3,2.9)	1.1(0.4,3.6)	38/1402(2.7)	5.3(3.4,8.5)	4.1(2.4,7.1)**
≥ 46	4/490 (0.8)	1	1	15/490(3.1)	6.1(3.3,11.2)	4.6(2.3,9.1)**
Occupation						
Farmer	10/1081(0.9)	1.1(0.1,8.6)	0.9(0.1,7.4)	22/1081(2.0)	1.4(0.3,5.8)	1.4(0.3,6.2)
Military	12/1956(0.6)	0.9(0.1,6.9)	1.1(0.1,8.9)	40/1956(2.0)	1.4(0.3,5.7)	0.6(0.1,2.5)
Government employed	11/1896(0.6)	0.6(0.8,4.8)	1.6(0.2,12.9)	31/1896(1.6)	1.1(0.3,4.6)	0.5(0.1,2.1)
Driver	1/222(0.5)	0.5(0.03,8.5)	1.9(0.1,31.4)	1/222(0.5)	0.3(0.03,3.3)	0.3(0.02,2.8)
Student	12/4079(0.3)	0.6(0.7,4.5)	1.8(0.2,14.5)	14/4079(0.3)	0.2(0.05,1.0)	0.2(0.04,0.8)**
Private employed	12/1259(1.0)	1.1(0.1,8.3)	0.9(0.1,7.4)	5/1259(0.4)	0.7(0.2,3.3)	0.2(0.03,0.9)**
Others*	4/757(0.5)	0.5(0.1,4.7)	1.9(0.2,17.9)	8/757(1.1)	3.9(0.7,20.1)	0.4(0.08,2.0)
Unemployed	1/132(0.8)	1		2/132(1.5)	1	1
Donor type						
Mobile	29/6376(0.5)	0.7(0.3,1.7)		82/6376(1.3)	0.9(0.5,1.6)	1.3(0.7,2.5)
Replacement	28/4089(0.7)	1.1(0.4,2.5)		28/4089(0.7)	0.5(0.3,0.9)	0.3(1.6,6.7)**
Voluntary	6/917(0.7)	1		13/917(1.4)	1	1

^*^Others: merchant, teacher, NGO, daily laborer, factory and housewife

^**^To indicate stastical significant

Looking at the association of predictor variables with viral hepatitis (HBV and HCV), males were two times more likely (AOR: 1.9; 95% CI: 1.4, 2.5) to be infected with HBV than females. Besides, government employees (AOR: 0.4; 95 CI: 0.2, 0.7) and students (AOR: 0.4; 95% CI: 0.2, 0.8) were 60% less likely to be infected than non-employees. While, those blood donors in the age group of ≥ 46 years (1.8%) were more than two times more likely to be infected with HCV than in the age group of 17–25 years (0.6%) (AOR: 2.7; 95 CI: 1.2, 6.2) (Table 5).

**Table 5:** Characteristics of blood donors associated with Hepatitis B and C virus seropositivity from 2008 to 2015 in Harari regional state blood bank in Eastern Ethiopia

Characteristics	Hepatitis B positive No (%)	Crude odd ratio 95% CI	Adjusted odds ratio 95% CI	Hepatitis C positive No (%)	Crude odd ratio 95% CI	Adjusted odds ratio 95% CI
Sex						
Male	448/9403 (4.8)	1.9(1.4,2.5)	1.7(1.3,2.3)**	80/9403(0.9)	1.4(0.8,2.5)	1.1(0.6,2.2)
Female	52/1979 (2.6)	1	1	12/1979(0.6)	1	1
Age						
17–25	267/6555 (4.1)	0.9(0.6,1.3)		40/6555(0.6)	1	1
26–35	144/2934(4.9)	1.1(0.7,1.6)		28/2934(1.0)	1.6(1.0,2.6)	1.5(0.8,2.7)
36–45	66/1402(4.7)	1.0(0.6,1.6)		15/1402(1.1)	1.8(1.0,3.2)	1.6(0.8,3.2)
≥ 46	23/490(4.7)	1		9/490(1.8)	3.1(1.5,6.3)	2.7(1.2,6.2)**
Occupation						
Farmer	57/1081(5.3)	0.6(0.3,1.2)	0.6(0.3,1.1)	15/1081(1.4)	1.8(0.8,3.9)	1.5(0.7,3.5)
Military	104/1956(5.3)	0.6(0.3,1.2)	0.6(0.3,1.1)	17/1956 (0.9)	1.1(0.5,2.4)	1.3(0.5,3.0)
Government employed	64/1896(3.4)	0.4(0.2,0.8)	0.4(0.2,0.7)**	12/1896(0.6)	0.8(0.3,1.8)	0.8(0.3,1.9)
Driver	11/222(5.0)	0.6(0.2,1.4)	0.5(0.2,1.3)	3/222(1.4)	1.7(0.5,6.3)	1.7(0.5,6.4)
Student	144/4079(3.5)	0.4(0.2,0.8)	0.4(0.2, 0.8)**	24/4079(0.6)	0.7(0.4,1.6)	1.1(0.5,2.7)
Private employed	59/1259(4.7)	0.5(0.3,1.1)	0.5(0.3,1.0)	10/1259(0.8)	1	
Others^*^	50/757(6.6)	0.8(0.4,1.5)	0.8(0.4,1.6)	11/889(1.2)	1.6(0.7,3.7)	1.5(0.7,3.7)
Unemployed	11/132(8.3)	1	1		1	1
Donor type						
Mobile	251/6376(3.9)	0.9(0.6,1.2)		44/6376(0.7)	1.1(0.5,2.5)	1.3(0.5,3.1)
Replacement	207/4089(5.1)	1.1(0.8,1.6)		42/4089(1.0)	1.6(0.7,3.7)	1.4(0.6,3.4)
Voluntary	42/917(4.6)	1		6/917(0.7)	1	1

^*^others: merchant, teacher, NGO, daily laborer, factory, housewife and unemployed

^**^To indicate statistical significant

## Discussion

The overall seroprevalence of transfusion transmitted infection was 6.6% in this study. This result did not include TTIs during the window period, which is serological negative. This still pose a threat to blood safety in environments. Thus, there might be higher rate of transfusion-transmissible infection in the community. However, the current study finding was higher than report from Eritrea (3.8%) [24] but lower than studies conducted in Jigjiga, eastern Ethiopia (11.5%) [25], Gondar, Ethiopia (9.5%) [19] and Sudan (13.1%) [26]. The difference might be due to difference in study area, study period (as there might change in the awareness of donors), socio demography of the study participants, strength of preliminary screening of donors and method of laboratory diagnosis used for screening of blood.

Majority of TTIs occurred in this study in the first time donors. This is similar to other study [19]. This can be due to replacement or mobile donors who are less likely to express about previous exposure and pass the primary screening and donate blood. In other words, there is low number of voluntary donors who seem to have low risk. There are a number of studies which indicate the prevalence rates of transfusion-transmissible infections that are found to be higher among replacement donors than voluntary donors [8, 9, 24]. These imply there is need on increasing the number of voluntary donor through creating awareness among the population. This might gradually abolish the replacement or mobile donations; thereby ensuring the safety of blood transfusion. However, in this study area, there is low number of voluntary donors. Thus, blood bank made campaigns in community, school, universities and other institution in order to increase number of blood donors in (personal communication from blood bank). This donor might have some risk of TTIS. There is need for rigorous screening. Otherwise this might increase the number unsafe blood to be disposed which can increases costs laboratory examination and disposal.

The highest TTI in this study was HBV (4.4%). This is slightly lower than in Gondar, Ethiopia (4.7%) [19]. This was lower than report of a study conducted in Jigjiga (10.9%) [26] and in Bahir Dar Hospital (6%) [21] in Ethiopia and other African countries like Tanzania (8.8%) [11] and Congo, Kinshasa (5.4%) [13]. This result is higher than a report from Eritrea (2.58%) [24] and Dessie and Mekelle, Ethiopia (3%) [21]. The difference might due to difference risky behaviors at different, geographical location, capacity of the primary screening test, method laboratory diagnostic and other. In this study, all laboratory tests to screen TTIs are based on ELISA principles which have potentially high sensitivity and specificity. Males were more likely to be infected with HBV. This is similar to study conducted in Gondar [19] and in Jigjiga [26], Ethiopia. This difference prevalence might be due to sex differences in behavioral risk factors such as having multiple sex partners and alcohol drinking. However, government employed and students were less likely to be infected with HBV than unemployed. This was different compared to study conducted in Gondar [19] which found farmers were more likely to be infected with HBV. The difference might be due to difference in awareness of individuals about HBV or difference in exposure practices.

Hepatitis B Virus infection was the most common reason for donor disqualification from donating blood in this study. This is similar to studies conducted elsewhere [8, 9, 11, 12, 27]. The current prevalence of HBV was categorized the study area as high intermediate endemic transmission area [28]. Hepatitis B Virus infection is known as the most serious blood borne pathogen which can cause chronic infection resulting in cirrhosis of the liver, liver cancer, liver failure and death. Persons with chronic infection can also remain a carrier for HBV transmission [29]. Thus, there is need for further assessment of potential risk factors in the community and strengthening the preventive measures like vaccine in order to prevent further transmission.

The second most TTIs in this study were syphilis (1.1%). This was higher than a report from Eritrea (0.49%) [24] and Jigjiga, Ethiopia (0.1%) [26]. This was slightly lower than study conducted in Gondar, Ethiopia (1.7%) [19], Tanzania (4.7%) [11] and Congo, Kinshasa (3.7%) [13]. In this study the prevalence syphilis is more likely to be increased with age. This is similar to report from Tanzania [24]. But it is not consistent with the study conducted in Gondar [19] and Jigjiga [26], Ethiopia. A similar higher prevalence HCV was detected in those study participants in the age group ≥ 46 in this study. Thus there is need to assess potential risk factors of syphilis and HCV among this higher age groups. Those, students were less likely to be infected with syphilis in this study. This is similar to report from Gondar [19]. The main reasons might be due student might acquire information about sexual transmitted infection through their school and might follow different prevention methods of sexually transmitted infection.

Hepatitis B virus and Syphilis area the most common coinfection in this study. However, HIV –syphilis and HIV-HBV were the most common co infection detected in Gondar study [19]. The difference might be due decrease in the prevalence of HIV through different effort. The above overlap in co infection might indicate, they are following similar transmission.

The seroprevalence of HIV was detected at 0.6% in this study which was lower than 2.8% reported in the general population in the Harari Region [29]. This was higher than a report from blood donors in Jigjiga (0.1%) [26] and Eretria (0.18%) [24]. But, it was lower than a report from Gondar, Ethiopia by Diro et al. (4.5%) [20] and Tessema et al. (3.8%) [19] and other studies conducted in Tanzania (3.8%) [11], Congo, Kinshasa (4.7%) [13]. The basic difference might be due to; difference in prevention measures taken and their effectiveness in different geographical location; risk of transmission; awareness peoples about HIV transmission and preventions.

In general, the prevalence of HCV and syphilis decline significantly. The prevalence of HBV and HIV decline, but the decline was not statistical significant in this study. However, significantly declining trends of HIV, HCV and syphilis seropositivity were observed in the Gondar study [19]. The overall decline in TTs in this study was not significant. Thus, there is need for more intervention on screening and other measures on the blood donors and the community for further the reduction all TTIs transmission in community.

This study has some limitations. It is a retrospective blood donation card review which might not include some variables. All test result did not give positive serological result during the window period. But, detailed preliminary risk factors assessment was made by trained health professionals in the blood bank unit before donation based on blood donors screening guideline. The method laboratory analysis doesn’t include molecular analysis which is more confirmatory test. However, this study tried to give better information on magnitude, trends and some associated factors of TTIs since it used large sample size and long year of blood donors’ history and laboratory tests records retrieved.

## Conclusion

The magnitude TTIs was lower than the previous studies conducted in Ethiopia. However, the study area has high intermediate endemic transmission. Majority of TTIS occurred among first time blood donors. Those students, private employed and government employed were less likely to be infected with syphilis and hepatitis B virus. Male were more likely to infected with HBV. There was significantly decline in the prevalence of HCV and Syphilis infection, but not for HIV and HBV. The prevalence of syphilis and HCV also increases with age. Therefore, strict adherence with the criteria of preliminary blood donor selection should be implemented to reduce the amount of blood being withdrawn from transfusion after collection and screening. It is also important to increase the number of repeated voluntary donors through promotion of blood bank activity. In addition, further study should be conducted to identify the gaps in the failure of preliminary screening in removing the donor before blood donation and feasible way increasing voluntary donors. There is also an assessment and taking measures on the potential risk factors of major TTI in the community.

## Abbreviations

BTS: Blood Transfusion Services
HBV: Hepatitis B Viruses
HCV: Hepatitis C Viruses
HIV: Human Immunodeficiency Virus
TTIs: Transfusion Transmitted Infections
WHO: World Health Organization
